# The Epstein-Barr Virus EBNA1 Protein

**DOI:** 10.6064/2012/438204

**Published:** 2012-12-19

**Authors:** Lori Frappier

**Affiliations:** Department of Molecular Genetics, University of Toronto, 1 Kings College Circle, Toronto, ON, Canada M5S 1A8

## Abstract

Epstein-Barr virus (EBV) is a widespread human herpes virus that immortalizes cells as part of its latent infection and is a causative agent in the development of several types of lymphomas and carcinomas. Replication and stable persistence of the EBV genomes in latent infection require the viral EBNA1 protein, which binds specific DNA sequences in the viral DNA. While the roles of EBNA1 were initially thought to be limited to effects on the viral genomes, more recently EBNA1 has been found to have multiple effects on cellular proteins and pathways that may also be important for viral persistence. In addition, a role for EBNA1 in lytic infection has been recently identified. The multiple roles of EBNA1 in EBV infection are the subject of this paper.

## 1. Introduction

 Epstein-Barr nuclear antigen 1 (EBNA1) was the first Epstein-Barr virus (EBV) protein detected and is the most widely studied [1]. EBNA1 is expressed in both latent and lytic modes of EBV infection, although it has mainly been studied in latency, where it plays multiple important roles. The importance of EBNA1 in EBV latency is reflected in the fact that EBNA1 is the only viral protein expressed in all forms of latency in proliferating cells and in all EBV-associated tumours. EBNA1 is required for the persistence of EBV genomes due to its contributions to both the replication and mitotic segregation of EBV episomes. EBNA1 also activates the expression of other EBV latency genes important for cell immortalization. All of these functions involve EBNA1 binding to specific DNA recognition sites in the EBV latent origin of DNA replication (*oriP*). In addition, EBNA1 has been shown to alter the cellular environment in multiple ways that might facilitate viral infection and contribute to cell immortalization and survival. This paper will review the known functions, cellular effects, and mechanisms of action of EBNA1.

## 2. EBNA1 Functions at the EBV Genomes

### 2.1. DNA Replication

The origin of latent DNA replication, termed *oriP* (for plasmid origin), was initially identified by screening EBV DNA fragments for the ability to enable the replication and stable maintenance of plasmids in human cells that were latently infected with EBV [2]. Subsequent studies showed that the only viral protein required for the replication of *oriP* plasmids was EBNA1 [3]. Both EBV episomes and *oriP* plasmids were found to replicate once per cell cycle, mimicking cellular replication and providing a good model system for human DNA replication [4, 5].

**Figure 1:**
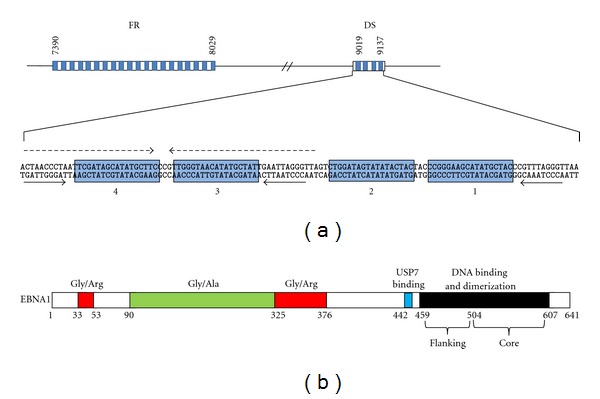
Schematic representation of *oriP* and the EBNA1 protein. (a) Organization of the *oriP* DS and FR elements showing genome nucleotide coordinates and EBNA1 binding sites (blue boxes). For the DS element, the positions of the four EBNA1-binding sites (numbered blue boxes), 65 bp dyad symmetry sequence (broken arrows), and nonamer repeats (solid arrows) are indicated. (b) Organization of the EBNA1 protein showing the two Gly-Arg-rich regions (red), Gly-Ala repeat (green), USP7-binding site (blue), and DNA binding and dimerization domain (black). Amino acids numbers are indicated below.

*OriP* is comprised of two functional components, the dyad symmetry (DS) element and the family of repeats (FRs) [6] (Figure 1(a)). The DS element contains four EBNA1 recognition sites, two of which are located within a 65 bp dyad symmetry sequence [6, 7]. Three copies of a 9 bp sequence, referred to as nonamers, were also identified at the ends and in the middle of the DS element (Figure 1(a)), [8] and were later shown to be binding sites for telomeric repeat binding factor 2 (TRF2) [9]. The FR element consists of 20 tandem copies of a 30 bp sequence, each of which contains an 18 bp palindromic EBNA1 binding site, followed by a 12 bp AT-rich sequence [6, 7]. The primary function of the FR is not in DNA replication but rather in the mitotic segregation and transcriptional activation functions of EBNA1 as discussed later. However the EBNA1-bound FR element can affect DNA replication by inhibiting the passage of replication forks, forming a major pause site [10–14].

The DS element is the origin of replication within *oriP* [10] and has been shown to be both essential and sufficient for plasmid replication in the presence of EBNA1 [15–17]. Efficient replication from the DS element requires all four EBNA1 binding sites as well as the nonamer repeats that flank the EBNA1 sites [18, 19]. A low level of DNA replication can be achieved with only two of the adjacent EBNA1 sites (either site 1 + 2 or site 3 + 4) but the 3 bp spacing between these sites is critical [16–18, 20, 21]. 

The EBNA1 DNA binding domain is essential for the replication function of EBNA1; however it is not sufficient for this activity, as the N-terminal half of EBNA1 is also required [22–29]. The replication function has not been disrupted by any single localized deletion or mutation and appears to involve redundant contributions of at least two EBNA1 regions (amino acids 8–67 and 325–376; Figure 1(b)). However, deletion of EBNA1 residues 61–83 or 395–450 has been observed to increase replication efficiency [29, 30], and, consistent with these results, a point mutation within each of these regions (G81 and G425) causes a similar enhancement of EBNA1-dependent DNA replication [31]. These point mutations disrupt EBNA1 binding to tankyrase, suggesting that tankyrase negatively regulates replication by EBNA1, possibly through the poly-ADP ribosylation of EBNA1 [31]. The EBNA1 395–450 region mediates an interaction with the host ubiquitin specific protease, USP7, suggesting that USP7 may negatively regulate replication [30].

EBNA1 is the only EBV protein involved in latent-phase DNA replication, but lacks any enzymatic activities such as DNA helicase or origin melting activities possessed by some viral origin binding proteins [32]. Therefore EBV depends heavily on host cellular proteins to replicate its episomes. Several studies have shown that the cellular origin recognition complex (ORC) and minichromosome maintenance (MCM) complex are associated with the DS element of *oriP*, implicating them in the initiation and licensing of EBV DNA replication [33–35]. In addition, a functional role for ORC in *oriP* plasmid replication was indicated by the failure of these plasmids to stably replicate in a cell line containing a hypomorphic *ORC2* mutation [35]. EBV replication was also found to be inhibited by geminin, a protein that inhibits rereplication from cellular origins by interacting with Cdt1 [35]. This suggests that Cdt1 loads the MCM complexes on EBV origins, as it does on cellular origins. 

EBNA1 has been shown to be important for ORC recruitment to the DS [33, 35, 36]. In addition, EBNA1 was recently found to interact with Cdc6, and this interaction increased ORC recruitment to the DS *in vitro* [37]. Interestingly, ORC is not recruited by EBNA1 bound to the FR element, suggesting that the DS DNA sequence or arrangement of the EBNA1 binding sites is important for ORC recruitment [33–35, 37]. ORC recruitment by EBNA1 requires EBNA1 N-terminal sequences including the Gly-Arg-rich regions (Figure 1(b)), [37, 38]. *In vitro*, these regions were found to interact with ORC through RNA molecules [38], although a second study suggested that, in the presence of Cdc6, EBNA1 could recruit ORC to the DS in an RNA-independent manner [37]. EBNA1 may also facilitate the recruitment of telomere repeat binding factor 2 (TRF2) to the three nonamer repeats of the DS, which contributes to origin activation [9, 37, 39]. TRF2 then appears to contribute to ORC recruitment to the DS in conjunction with EBNA1 [20, 36] and also affects the timing of replication in S phase through recruitment of additional proteins [40, 41]. 

In addition, EBNA1 has been found to recruit template activating factor I*β* (TAF-I*β* also called SET) to both the DS and FR elements, through a direct interaction with the 325–376 Gly-Arg-rich region of EBNA1 [42, 43]. TAF-I*β* negatively regulates replication from *oriP *as TAF-I*β* depletion was found to increase *oriP *plasmid replication while TAF-I*β* overexpression inhibited it [43]. Since TAF-I*β* is a nucleosome-associated protein that can recruit either histone acetylases or deacetylases [44, 45], TAF-I*β* may negatively regulate replication from *oriP* by affecting the chromatin structure.

### 2.2. Mitotic Segregation

EBV episomes are present at low copy numbers and replicate only once per cell cycle. Therefore their maintenance at a stable copy number in dividing cells requires a mechanism to ensure even segregation during cell division. The mitotic segregation or partitioning of the EBV episomes requires two viral components, EBNA1 and the *oriP* FR element [46–48]. In fact, EBNA1 and the FR element confer stability on a variety of constructs even when combined with heterologous origin sequences [47, 49, 50]. EBNA1 binding to its multiple recognition sites in the FR is crucial for its segregation function, as is the central Gly-Arg-rich region of EBNA1 (325–376; Figure 1(b)), [27]. 

EBNA1 functions in segregation by tethering the EBV episomes to the cellular chromosomes in mitosis. Accordingly, EBNA1 and EBV episomes and *oriP*-containing constructs have all been found to associate with mitotic chromosomes [50–54], and the association of *oriP* plasmids with mitotic chromosomes was shown to depend on the EBNA1-chromosome association [55, 56]. In addition, EBNA1 mutants that are nuclear but defective in mitotic chromosome attachment fail to partition *oriP *plasmids [27, 57, 58]. EBNA1 and EBV episomes are not localized to particular regions of mitotic chromosomes, but rather are widely distributed over the chromosomes, leading to the initial suggestion that EBNA1 and EBV episomes interact randomly with chromosomes [51]. However, subsequent studies have indicated that initial pairing of EBV episomes on sister chromatids may ensure their equal distribution to the daughter cells and that this pairing may stem from the catenation of the newly replicated EBV plasmids [52, 59–61]. In addition, the FR element has been found to direct EBV genomes to chromatin regions with histone modifications typical of active chromatin [62].

Studies with EBNA1 deletion mutants showed that the central Gly-Arg-rich region of EBNA1 (amino acids 325–376) was critical for chromosome attachment and that N-terminal sequences (8–67) also contribute to this interaction [27, 29, 57, 58, 63, 64]. Interestingly, fusion proteins in which these EBNA1 regions have been replaced by other chromosome binding sequences are also able to support *oriP* plasmid maintenance [57, 65]. Both the central Gly-Arg repeat of EBNA1 and sequences spanning the smaller Gly-Arg-rich N-terminal sequence (amino acids 33–53; Figure 1(b)) can cause proteins to associate with mitotic chromosomes when fused to them [57, 63, 66]. However, deletion of the N-terminal Gly-Arg sequence within EBNA1 does not affect EBNA1's ability to maintain *oriP* plasmids or to associate with mitotic chromosomes, indicating that it is the central Gly-Arg-rich region that is normally used by EBNA1 for chromosome interactions and segregation [29, 67]. This region contains a repeated GGRGRGGS sequences that is phosphorylated on the serines and methylated by PRMT1 or PRMT5 on the arginine residues [64, 68].

The segregation of viral genomes by attachment to cellular chromosomes is not unique to EBV but is a strategy also used by Kaposi sarcoma-associated herpesvirus (KSHV) and papillomavirus. In each case the viral origin binding (LANA for KSHV and E2 for papillomavirus) tethers the viral plasmid to the cellular chromosome through interactions with one or more cellular proteins [69–72]. For EBNA1, interactions with the cellular protein, EBP2, appears to be important for metaphase chromosome attachment and segregation function [27, 58, 73, 74]. The EBNA1 325–376 region critical for chromosome attachment also mediates EBP2 binding, and there is a close correspondence between the effect of EBNA1 mutations on EBP2 and metaphase chromosome interactions [27, 29, 58, 64, 67]. In addition, EBP2 depletion in various cell lines, including the EBV-positive C666-1 NPC cells, resulted in redistribution of EBNA1 from the metaphase chromosomes to the soluble cell fraction and a corresponding release of *oriP* plasmids from the chromosomes [56]. EBP2 was also found to enable EBNA1-mediated plasmid segregation in budding yeast by facilitating EBNA1 attachment to the yeast mitotic chromosomes [49, 73]. 

 However, examination of the timing of chromosome association in human cells showed that EBNA1 associates with the chromosomes earlier in mitosis than EBP2 and that EBNA1 and EBP2 only associate on the chromosomes in metaphase to telophase [67]. This suggests that EBNA1 initially contacts the chromosomes by an EBP2-independent mechanism and that subsequent interactions with EBP2 in mid-to-late mitosis might be important to maintain EBNA1 on chromosomes. The initial chromosome contact might involve direct DNA binding or interactions with chromosome-associated RNA molecules, since the 325–376 and N-terminal arginine-rich regions have been found to have some capacity to interact with DNA and RNA *in vitro*, and drugs that bind G-quadruplex RNA have been reported to decrease the mitotic chromosome association of EBNA1 [38, 66, 75, 76]. Recently FRET analysis identified an interaction between EBNA1 and EBP2 in the nucleoplasm and nucleolus in interphase suggesting additional roles for this interaction, including the possibility that the EBNA1-EBP2 interaction in interphase is important for EBNA1-chromosome interactions in mitosis [77]. This is intriguing in light of the findings that the E2 papillomavirus protein requires an interphase interaction with host ChlR1 in order to associate with mitotic chromosomes to segregate papillomavirus genomes [72, 78].

### 2.3. EBV Transcriptional Activation

EBNA1 can also act as a transcriptional activator when bound to the *oriP* FR element, enhancing the expression of reporter genes on FR-containing plasmids in a distance-independent manner [46, 79]. The EBNA1-bound FR was also shown to activate expression from the viral Cp and LMP promoters, suggesting a role for EBNA1 in inducing the expression of the EBNA and LMP EBV latency genes in latent infection [80, 81]. The EBNA1 residues required for transcriptional activation have been mapped to the central Gly-Arg-rich region (residues 325–376) also required for segregation function [22, 23, 28, 82] and to the 61–89 N-terminal sequence [29, 83]. EBNA1 requires both of these regions to activate transcription as deletion of either one abrogates the transcriptional activation function of EBNA1 [28, 29]. A Δ61–83 EBNA1 mutant was found to be fully active for replication and segregation functions, indicating that transcriptional activation is a distinct EBNA1 function [29]. Similar conclusions were reached with a Δ65–89 EBNA1 mutant in the context of an infectious EBV; where EBNA1 Δ65–89 was shown to be defective in activating expression of the EBNA genes from the Cp promoter, but still supported stable plasmid replication [84]. EBV containing the Δ65–89 EBNA1 was also shown to be severely impaired in the ability to transform cells, indicating the importance of EBNA1-mediated transcriptional activation for EBV infection [84]. 

The fact that two transcriptional activation sequences are required for efficient transcriptional activation by EBNA1 suggests that they make unique contributions to this process, likely by mediating different cellular protein interactions. The 61–83 region was found to mediate an interaction with Brd4 [85], a cellular bromodomain protein that interacts with chromatin to regulate transcription [86]. In addition, Brd4 preferentially localized to the EBNA1-bound FR enhancer element in EBV genomes and Brd4 depletion inhibited EBNA1-mediated transcriptional activation [85]. The results suggest that EBNA1 uses Brd4 to activate transcription. Interestingly, an interaction between Brd4 and papillomavirus E2 proteins (the functional equivalent to EBNA1) has been shown to be important for transcriptional activation by E2 [87–89], suggesting that EBNA1 and E2 may use common mechanisms to activate transcription. 

The EBNA1 325–376 region mediates interactions with several cellular proteins, some of which have been implicated in the transcriptional activity of EBNA1. For example, P32/TAP, which interacts with Arg-rich sequences, has been detected at *oriP* by chromatin immunoprecipitation, and its C-terminal region has some ability to activate a reporter gene when fused to the GAL4 DNA-binding domain [23, 82]. However, it is not clear whether P32/TAP is important for EBNA1-mediated transcriptional activation. The related nucleosome assembly proteins, NAP1 and TAF-I*β* (also called SET), also interact with the EBNA1 325–376 sequence and are known to affect transcription in multiple ways [30, 43, 90]. A role for NAP1 and TAF-I*β* in EBNA1-mediated transcriptional activation is supported by the finding that both proteins are recruited to the FR element by EBNA1 and that EBNA1 transactivation activity is decreased upon depleting either protein [43]. Interestingly, NAP1 has been shown to bind and enhance the transcriptional activation activity of the papillomavirus E2 protein, through recruitment of the p300 histone acetyltransferase [91]. This suggests that, like Brd4, NAP1 is used by both EBNA1 and E2 for transcriptional activation. 

Transcriptional activation by EBNA1 may not only be influenced by histone acetylation but also by ubiquitylation of histone H2B. The latter is suggested by the finding that EBNA1 binds to a complex of USP7 and GMP synthetase, that functions to deubiquitylate H2B and recruits it to the FR [92]. USP7 depletion results in increased levels of monoubiquitylated H2B at the FR and decreased transcriptional activation, suggesting that monoubiquitylation of H2B inhibits EBNA1-mediated transcriptional activation. In keeping with this result, an EBNA1 mutant defective in USP7 binding was found to have decreased transcriptional activation activity [30].

### 2.4. Autoregulation

In addition to interactions with the *oriP* FR and DS elements, EBNA1 was found to bind a third region of the EBV genome near the Qp promoter that is used to express EBNA1 in the absence of other EBNAs [93–95]. EBNA1 binding to two recognition sites located downstream of Qp was reported to repress EBNA1 expression from Qp [94]. Since EBNA1 has lower affinity for these sites than either the DS or FR elements, EBNA1 would only bind the Qp sites when its levels are high enough to saturate the FR and DS elements, providing a feedback mechanism to shut off EBNA1 expression when EBNA1 levels are high [93, 96]. While EBNA1 was initially thought to inhibit expression from Qp by repressing transcription, a more recent study found that EBNA1 acts post- or cotranscriptionally to inhibit the processing of primary transcripts [97]. 

## 3. EBNA1-DNA Interactions

### 3.1. Interactions with the EBV Genome

EBNA1 is a DNA binding protein that specifically interacts with three regions of the EBV genome, the *oriP* FR and DS elements, and the BamHI-Q fragment containing the Qp promoter [7, 93]. All of the EBNA1-bound EBV fragments contain multiple copies of an 18 bp palindromic sequence that was protected by EBNA1 in DNA footprints [7, 96]. The multiple copies of this sequence in the EBV genome contain some sequence variations that account for the different affinities of EBNA1 for the FR, DS, and BamHI-Q regions and for individual sites within these regions [96, 98]. EBNA1 has highest affinity for the FR and DS regions and remains bound to these sites throughout the cell cycle [8, 99, 100]. 

EBNA1 interacts with its recognition sites through its C-terminal domain (amino acids 459 and 607; Figure 1(b)), which also mediates the dimerization of EBNA1 [98, 101–103]. EBNA1 forms very stable homodimers both in solution and when bound to its recognition sites [32, 101, 103]. The crystal structure of the DNA binding and dimerization domain was determined both in solution and bound to the EBNA1 consensus binding site [104, 105]. The structure revealed that dimerization was mediated by residues 504–604 (referred to as the core domain), which form an eight-stranded antiparallel *β*-barrel, comprised of four strands from each monomer and two *α*-helices per monomer. This core domain is strikingly similar to the structure of the DNA-binding domain of the E2 protein of papillomavirus, despite a complete lack of sequence homology [106, 107]. Residues 461–503 flank the core domain (flanking domain) and are comprised of an *α*-helix oriented perpendicular to the DNA and an extended chain that tunnels along the base of the minor groove of the DNA. Both the helix and the extended chain make sequence-specific DNA contacts. In addition, a direct role of the core domain in DNA recognition was suggested by analogy to the E2 DNA-binding domain and later confirmed by mutational analyses [108]. Combined, the structural and biochemical studies indicate that the core and flanking domains of EBNA1 work together to load EBNA1 on its recognition site, likely through a two-step DNA-binding mechanism. In keeping with this model, thermodynamic and kinetic analyses of the EBNA1 DNA-binding domain-DNA interaction revealed two DNA association and dissociation events [109]. In addition, the ability of EBNA1 to bind its recognition sites, both *in vitro* and *in vivo*, was found to be greatly stimulated by USP7 through its interaction with EBNA1 amino acids close to the flanking domain (442–448; Figure 1(b)), [92], suggesting that this USP7 interaction may facilitate the DNA loading of the flanking domain. 

The interaction of the EBNA1 DNA-binding and dimerization domain with a single recognition site causes the DNA to be smoothly bent and causes localized regions of helical overwinding and underwinding [105]. The overwinding is caused by the EBNA1 flanking domain residues that traverse along the minor groove (amino acids 463–468) [110, 111], and this results in the increased sensitivity of one T residue within the DS sites to permanganate oxidation [99, 111–113]. EBNA1 dimers assemble cooperatively on adjacent sites in the DS [16, 98], and this is predicted to induce additional changes in the DNA structure (such as unwinding), in order to accommodate the closely packed dimers [105]. The strict requirement for the 3 bp spacing that separates neighboring sites in the DS for origin function suggests that the proper interaction between the EBNA1 dimers bound to these sites is crucial for the initiation of DNA replication, possibly because of the DNA structural changes that it imparts [16, 21]. Interactions of EBNA1 dimers on the multiple sites within the DS and FR elements likely also contribute to the pronounced bending of these elements that have been observed and to the appearance of EBNA1 as a large single complex on each element [21, 114, 115]. 

EBNA1 complexes bound to the DS and FR elements of *oriP* can also interact with each other cause the looping out of the intervening DNA (when interactions occur within an *oriP* molecule) and the linking of multiple *oriP* molecules (when interactions occur between *oriP* molecules) [114–117]. The DNA looping and linking interactions stabilize EBNA1 binding to the DS and involve homotypic interactions mediated by two different regions of EBNA1: a stable interaction mediated by amino acids 327–377 and a less stable interaction mediated by residues 40–89 [26, 68, 118–120]. The looping/linking interactions of EBNA1 are not restricted to EBNA1 complexes formed on the DS or FR elements but also occur between single EBNA1 dimers bound to distant recognition sites [115]. The contribution of DNA looping and linking to EBNA1 functions remains unclear but the amino acids required for these interactions overlap with those required for EBNA1 replication, segregation, and transcriptional activation functions [26, 27, 29].

*In vivo* EBV genomes are assembled into nucleosomes with a spacing similar to that in cellular chromatin [121]. Since nucleosomes tend to inhibit sequence-specific DNA interactions, the ability of EBNA1 to bind its site in the DS in the context of a nucleosome was examined. Surprisingly, EBNA1 was able to access its recognition sites within the nucleosome and destabilized the nucleosome structure such that the histones could be displaced from the DNA [122]. Efficient assembly of EBNA1 on the FR and DS elements was also observed on larger *oriP* templates containing physiologically spaced nucleosomes [123]. The disruption of the DS nucleosome by EBNA1 required all four recognition sites in the DS and was intrinsic to the DNA binding and dimerization domain of EBNA1 [122]. The ability of EBNA1 to destabilize nucleosomes might be important for initiating DNA replication, a process known to be sensitive to nucleosome positioning. In addition, the ability of EBNA1 to access its sites within a nucleosome is likely to be important at times when chromatin is established prior to EBNA1 expression, for example, when latently infected resting cells (which do not express EBNA1) switch to proliferating forms of latency in which EBNA1 is expressed.

### 3.2. Interactions with Cellular DNA Sequences

The fact that EBNA1 can activate transcription, when bound to the EBV FR element, has prompted several studies to determine if EBNA1 might also interact with specific sequences in cellular DNA to affect cellular gene expression. Chromatin IP (ChIP) experiments performed for EBNA1 from EBV-positive lymphoblastoid cell lines, followed by promoter array analysis, identified several EBNA1-associated DNA fragments, some of which were confirmed to be directly bound by EBNA1 *in vitro* [124]. While this approach identified a new EBNA1 recognition sequence (distinct from those in *oriP*), EBNA1 binding to this sequence did not activate reporter gene expression so the significance of these EBNA1-cellular DNA interactions is not clear. ChIP combined with deep sequencing was also used to determine EBNA1-binding sites in B cells, identifying many EBNA1-associated sites, several of which were close to transcriptional start sites for cellular genes [125]. The expression of some of these cellular genes was decreased upon EBNA1 depletion and induced by EBNA1 expression, suggesting that EBNA1 may affect their transcription. Like the previous study, these EBNA1 sites differed from those in *oriP*, but some were similar in sequence to those identified by Dresang et al. [124]. In addition, a cluster of high-affinity EBNA1 binding sites was identified on chromosome 11 between the divergent FAM55D and FAM55B genes, although the expression of these genes was not affected by EBNA1 [125]. Canaan et al. [126] conducted microarray experiments to compare cellular transcripts in B cells and 293 cells with and without EBNA1 and identified a small percentage of transcripts that were affected by EBNA1. In addition, EBNA1 was found to ChIP to most of these gene promoters, suggesting it directly regulated them. However, whether or not EBNA1 bound directly to these promoters was not determined, and it is not yet clear how the array of genes affected might contribute to EBV infection or associated cancers. 

The transcriptional activation function of EBNA1 on the EBV genome requires EBNA1 binding to multiple tandem recognition sites in the FR [15], and therefore it seems unlikely that EBNA1 binding to any single recognition site would be sufficient to activate cellular transcription. To increase the probability of identifying functionally relevant EBNA1 interactions with cellular DNA, d'Herouel et al. [127] used nearest neighbor position weight matrices to identify repeated EBNA1-binding sites in the human genome. The sites they identified had considerable overlap with those found by Dresang et al. [124]. Although the significance of the repeated EBNA1 sites that they identified remains to be determined, it is interesting that they include weak binding sites near the c-Jun and ATF promoters, which were previously shown to be activated by and associated with EBNA1 in NPC cells [128]. 

Finally, Lu et al. [129] identified survivin as an EBNA1 target gene by comparing cell cycle-specific transcripts from EBV-negative B cells with and without EBNA1 expression. EBNA1 increased the levels of survivin transcripts and protein and was subsequently shown to associate with the survivin promoter. Induction of survivin protein and transcripts required EBNA1 amino acids 65–89, which are known to be important for transcriptional activation of EBV genes [29, 83], suggesting that EBNA1 was activating the transcription of the survivin gene. However, EBNA1 may interact with the promoter through the Sp1-host protein, since activation of the survivin promoter by EBNA1 involves the Sp1-binding sites. 

Presumably any of the above direct interactions of EBNA1 with specific DNA sites would be mediated by the EBNA1 DNA-binding domain. However, it has been suggested that EBNA1 might also mediate less specific cellular DNA interactions through its Gly-Arg-rich regions, which resemble AT hooks [66]. Indeed, these regions have been shown to have some ability to interact with AT-rich DNA *in vitro*. However, these regions also interact with several cellular proteins, and it remains to be determined whether the Gly-Arg-rich regions directly contact DNA in cells.

## 4. Cellular Effects of EBNA1 

In addition to the roles of EBNA1 at the EBV genome, numerous reports suggest that EBNA1 directly contributes to cell proliferation and survival typical of latent EBV infection. The first implications came from the observations that EBNA1 is the only EBV protein expressed in all EBV-positive tumours and latency types in proliferating cells and is sometimes the only EBV protein expressed. EBNA1 was subsequently shown to be important for efficient B-cell immortalization by EBV [84, 130] and for the continued proliferation of some EBV-positive tumour cells [131–133]. EBNA1 expression in various EBV-negative cancer cells has also been found to increase tumorigenicity [134–137]. In addition, EBNA1 expression in the B-cell compartment of a transgenic mouse has been reported to be sufficient to induce B cell lymphomas [138, 139]. However, these results were not reiterated in a second independent transgenic mouse study, suggesting that secondary events might contribute to the development of EBNA1-induced lymphomas [140, 141]. Nonetheless, the body of evidence indicates that EBNA1 contributes to oncogenesis, likely due to multiple effects on cellular proteins as discussed in the following. 

### 4.1. USP7 Interaction

Proteomics methods (affinity column profiling and TAP tagging) identified several cellular proteins that are bound by EBNA1, including an interaction with the cellular ubiquitin-specific protease USP7 (also called HAUSP [30, 142]). USP7 was originally discovered as a binding partner of the ICP0 protein from herpes simplex virus type 1 and has since been shown to be targeted by proteins from several different herpes viruses [143–146]. USP7 has been reported to bind and regulate several cellular proteins including p53 and Mdm2 (an E3 ubiquitin ligase for p53), which USP7 stabilizes by removing the polyubiquitin chains that normally signal degradation [147–151]. EBNA1, p53, and Mdm2 compete for the same binding pocket in the N-terminal TRAF domain of USP7; however EBNA1 was found to outcompete p53 or Mdm2 due to its higher affinity for USP7 [152–154]. The EBNA1 region just N-terminal to the DNA binding domain was identified as the USP7-binding site, and a subsequent crystal structure of this EBNA1 peptide bound to the USP7 TRAF domain showed that EBNA1 amino acids 442–448 contact USP7 (Figure 1(b)) and bind USP7 residues in addition to those contacted by p53 or Mdm2 [152–155]. 

In theory, EBNA1 could destabilize either p53 or Mdm2 by blocking their interaction with USP7, resulting in opposite effects on p53 levels. *In vivo* EBNA1 has not been reported to lower Mdm2 levels, but has been confirmed to lower p53 levels at least in some cell backgrounds. For example, expression of EBNA1 but not a USP7-binding mutant of EBNA1 in U2OS cells was shown to reduce the accumulation of p53 in response to DNA damage and subsequent apoptosis [153]. Similarly, EBNA1 expression in CNE2 nasopharyngeal carcinoma (NPC) cells decreased the accumulation of p53 in response to DNA damage [156], and the presence of EBNA1 or EBV in AGS or SCM1 gastric carcinoma cells decreased the steady-state levels of p53 [135, 157]. This suggests that EBNA1 could promote cell survival by modulating p53 in EBV-infected epithelial cells.

### 4.2. Effects on PML Nuclear Bodies

Promyelocytic leukemia (PML) nuclear bodies (also called ND10s) are nuclear foci for which PML tumour suppressor proteins form the structural basis. PML bodies are important for several cellular processes, including apoptosis, DNA repair, senescence, and p53 activation by acetylation [158–163], and their loss has been associated with the development and/or progression of several tumours [158, 164]. In addition, PML nuclear bodies suppress lytic viral infection as part of the innate antiviral response [165–167]. To counter this defense, many viruses encode proteins that disrupt PML nuclear bodies either by interfering with the interactions of PML proteins to form the bodies or by inducing the degradation of the PML proteins [168]. 

EBNA1 was found to induce the loss of PML nuclear bodies in both NPC and gastric carcinoma cells, by inducing the degradation of the PML proteins [156, 157]. Consistent with the known PML functions, EBNA1 expression in these cells was also found to decrease DNA repair efficiency, p53 acetylation, and apoptosis in response to DNA damaging agents [156, 157]. The results suggest that, as a result of EBNA1-induced PML loss, cells expressing EBNA1 are more likely to survive with DNA damage, which would be expected to contribute to the development of carcinomas. Importantly, these observations in cell lines appear to hold true *in vivo*, as a comparison of EBV-positive and EBV-negative gastric carcinoma tumour biopsies showed that PML levels were greatly reduced by the presence of EBV, presumably due to the action of EBNA1 [157]. 

 The mechanism by which EBNA1 induces the degradation of PML proteins involves EBNA1 binding to both USP7 and the host casein kinase 2 (CK2) and recruitment of these proteins to the PML nuclear bodies [156, 169]. EBNA1 was found to preferentially interact with PML isoform IV over the other five nuclear PML isoforms, and therefore EBNA1 may localize to PML nuclear bodies through interactions with PML IV [156, 170]. EBNA1 mutants that fail to bind either USP7 or CK2 can still associate with PML bodies but do not induce their loss [156, 169]. Similarly, wildtype EBNA1 does not affect PML nuclear bodies when USP7 or CK2 is depleted. In keeping with these observations, USP7 was subsequently shown to negatively regulate PML proteins (even in the absence of EBV or EBNA1), by a mechanism that is independent of its ubiquitin cleavage activity [171]. 

The interaction of EBNA1 with CK2 involves a direct interaction with the *β* regulatory subunit of CK2 through EBNA1 amino acids 387–394 [169]. CK2 was previously identified as a negative regulator of PML and was shown to phosphorylate PML proteins at a particular serine residue that triggers polyubiquitylation and subsequent degradation [172, 173]. Through its interaction with CK2, EBNA1 was shown to increase CK2-mediated phosphorylation of PML, which would increase PML polyubiquitylation [169]. Since CK2 is involved in many cellular processes, it is possible that the interaction of EBNA1with CK2 also affects additional pathways.

### 4.3. Modulation of Signaling Pathways

EBNA1 has been reported to affect several signaling pathways. First, EBNA1 expression in three different carcinoma cell lines was found to increase the expression of STAT1 [174, 175]. EBNA1 was subsequently shown to enhance STAT1 phosphorylation and nuclear localization in response to IFN*γ* [174]. Second, EBNA1 expression was found to decrease the expression of TGF-*β*1-responsive genes suggesting that EBNA1 interferes with TGF-*β* signaling [174]. This effect may be due to increased turnover of SMAD2 in the presence of EBNA1, resulting in decreased levels of SMAD complexes needed for TGF-*β*1-induced transcription [174, 176]. Third, using NF-*κ*B reporter plasmids in carcinoma cell lines, EBNA1 was found to inhibit NF-*κ*B activity and DNA binding [177]. Additional experiments showed that the levels, nuclear localization, and phosphorylation of the p65 NF-*κ*B subunit were all reduced in the presence of EBNA1 as was the phosphorylation of the p65 kinase, IKK*α*/*β* [177]. How EBNA1 elicits any of the above effects is presently unclear as no physical interaction has been detected between EBNA1 and STAT1, SMAD2, p65, or IKK*α*/*β*. 

### 4.4. Induction of Oxidative Stress

EBV infection is associated with increased oxidative stress [178, 179], and this may be at least partly due to EBNA1 expression. Stable or transient EBNA1 expression in B-cell lines was found to increase levels of reactive oxygen species (ROS), DNA damage foci, and dysfunctional, uncapped telomeres, and these EBNA1 effects were decreased by ROS scavengers [180, 181]. In addition, EBNA1 was found to increase the expression of the NOX2 NADPH oxidase which might account for the ROS induction [180]. Similarly, a comparison of the nuclear proteome in NPC cells with and without EBNA expression showed that EBNA1 increased the levels of several oxidative stress response proteins including the antioxidants superoxide dismutase 1 and peroxiredoxin 1, known to be induced by ROS [182]. Further studies confirmed that, in the presence of EBNA1, ROS levels were elevated and that NOX1 and NOX2 transcripts were increased [182]. Therefore EBNA1 appears to have multiple effects on the oxidative stress response, although the mechanisms of these effects are not yet known.

### 4.5. Effects on Metastatic Potential

A comparison of the nuclear proteomes of NPC cells with and without EBNA1 expression found that EBNA1 increased the nuclear levels of Nm23-H1, stathmin 1, and maspin, all of which have been found to be contribute to metastases [182]. This effect on Nm23-H1 corroborated a previous study that showed that EBNA1 coimmunoprecipitated with Nm23-H1 from lymphoid cells and caused it to relocalize to the nucleus [183]. This interaction required EBNA1 amino acids 65–89, which are also important for transcriptional activation [183]. Nm23-H1 is a known suppressor of metastasis and cell migration, and EBNA1 was shown to counteract the ability of Nm23-H1 to suppress cell migration both *in vitro* and in a nude mouse model [137, 183]. The results suggest that EBNA1 contributes to the metastatic potential of EBV tumours and are supported by a report by Sheu et al. [134] that EBNA1 expression in HONE-1 NPC cells increased tumour metastases in nude mice. In addition, the relocalization of Nm23-H1 by EBNA1 may be another way that EBNA1 decreases apoptosis and increases cell proliferation, as pathway-specific microarray analysis recently suggested roles for Nm23-H1 in promoting apoptosis and inhibiting cell proliferation [184]. 

## 5. Immune Evasion

While most of the EBV latency proteins elicit a strong immune response, cells that only express EBNA1 (referred to as latency I or EBNA1-only program) largely avoid immune detection [185–187]. This is due to inefficient presentation of EBNA1 peptides on MHC class I molecules [188]. Reduced EBNA1 presentation has been attributed to the central Gly-Ala repeat of EBNA1 which varies in size in different EBV isolates (~230 amino acid long in the B95-8 strain; Figure 1(b)). Removal of this repeat has been shown to restore EBNA1 presentation while the addition of the Gly-Ala repeat to EBNA4 inhibits its recognition by cytotoxic T lymphocytes [188, 189]. 

Initially it was thought that the Gly-Ala repeat inhibited EBNA1 presentation by interfering with its proteasomal processing. This was based on studies in which insertion of the Gly-Ala repeat in other proteins inhibited their degradation [190–194]. However, the deletion of the Gly-Ala repeat from EBNA1 was not found to affect EBNA1 turnover, as EBNA1 is extremely stable with or without the Gly-Ala repeat [195]. In addition, Tellam et al. [196] found that EBNA1 turnover varied considerably in different cell backgrounds but that these rates did not correspond to the level of MHC I-restricted presentation of EBNA1 peptides. Moreover, EBNA1 presentation was shown to derive from newly synthesized protein, and the primary contribution of the Gly-Ala repeat on the presentation of EBNA1 peptides was found to be due to inhibition of its own translation [196, 197]. This supported a model where the MHC I-restricted presentation of EBNA1 occurs through the generation of defective ribosomal products (DRiPs) which are reduced by the presence of the Gly-Ala repeat [198]. Further studies confirmed that MHC class I presentation and CTL recognition corresponded to the rate of EBNA1 translation and the levels of DRiPs, and that all were inhibited by the Gly-Ala repeat [199–201]. The Gly-Ala repeat was further shown to interfere with the initiation stage of translation [200]. In addition, EBNA1 presentation was shown to depend on the rate at which the DriPs were generated, rather than on their degradation [199], a phenomenon that involves purine loading of the EBNA1 mRNA [202]. Exactly how the Gly-Ala repeat regulates translation initiation is not yet clear but Hsp90 has been reported to be a contributing factor [203].

## 6. EBNA1 in Lytic EBV Infection

The above roles of EBNA1 all pertain to latent EBV infection. However, EBNA1 is also expressed in lytic infection from a lytic cycle-specific promoter (Fp), suggesting that it also contributes to productive infection [204–206]. Recently the contributions of EBNA1 to viral reactivation to the lytic cycle were examined in EBV-positive AGS gastric carcinoma cells [170]. EBNA1 silencing was found to increase the frequency with which EBV spontaneously entered the lytic cycle, suggesting that EBNA1 can suppress reactivation. In contrast, when the lytic cycle was chemically induced, EBNA1 silencing inhibited lytic gene expression and viral genome amplification indicating that EBNA1 can promote lytic infection once the lytic switch has occurred. However EBNA1 did not positively contribute to lytic infection when the PML proteins were silenced, suggesting that the role of EBNA1 in lytic infection was in overcoming suppression by PML proteins. In keeping with this interpretation, PML proteins and nuclear bodies were found to suppress lytic infection by EBV [170, 207]. 

## 7. Conclusion

In summary, EBNA1 makes multiple contributions to EBV infection due to its ability to interact with specific DNA sequences and multiple cellular proteins. Latency contributions include the replication and mitotic segregation of EBV episomes, contributions to viral transcription, and multiple effects on cellular proteins and pathways that promote cell survival and proliferation. Several of the cellular changes also result in increased DNA damage which could contribute to the development of EBV-associated tumours. Finally, EBNA1 is now known to have a role in lytic infection in overcoming suppression by PML nuclear bodies, further emphasizing its importance in EBV infection.
